# Clinical Management of Cerebral Amyloid Angiopathy

**DOI:** 10.3390/jcm14124259

**Published:** 2025-06-15

**Authors:** Aikaterini Theodorou, Stella Fanouraki, Eleni Bakola, Georgia Papagiannopoulou, Lina Palaiodimou, Maria Chondrogianni, Maria-Ioanna Stefanou, Lampis Stavrinou, Athanasia Athanasaki, Klearchos Psychogios, Odysseas Kargiotis, Apostolos Safouris, Georgios Velonakis, Georgios P. Paraskevas, Georgios Tsivgoulis

**Affiliations:** 1 Second Department of Neurology, “Attikon” University Hospital, School of Medicine, National and Kapodistrian University of Athens, Rimini 1, Chaidari, 12462 Athens, Greece; katetheo24@gmail.com (A.T.); stelfanou@gmail.com (S.F.); georgiapap22@hotmail.com (G.P.); mariachondrogianni@hotmail.gr (M.C.); marianna421@hotmail.co.uk (M.-I.S.); athanasia.athan@yahoo.gr (A.A.); safouris@yahoo.com (A.S.); geoprskvs44@gmail.com (G.P.P.); 2 Department of Neurology & Stroke, Eberhard-Karls University of Tübingen, 72074 Tübingen, Germany; 3 Hertie Institute for Clinical Brain Research, Eberhard-Karls University of Tübingen, 72074 Tübingen, Germany; 4 Second Department of Neurosurgery, “Attikon” University Hospital, School of Medicine, National and Kapodistrian University of Athens, 12462 Athens, Greece; lampis.stavrinou@gmail.com; 5 Stroke Unit, Metropolitan Hospital, 18547 Piraeus, Greece; apsychoyio@yahoo.gr (K.P.); kargiody@gmail.com (O.K.); 6 Second Department of Radiology, “Attikon” University Hospital, School of Medicine, National and Kapodistrian University of Athens, 12462 Athens, Greece; giorvelonakis@gmail.com

**Keywords:** cerebral amyloid angiopathy, cerebral microbleeds, cortical superficial siderosis, CAA-ri, amyloid-β, brain MRI, cerebrospinal fluid biomarkers, amyloid PET, treatment

## Abstract

**Background:** Cerebral amyloid angiopathy (CAA) represents a progressive cerebrovascular disorder, characterized by aberrant accumulation of beta-amyloid isoforms in cortical and leptomeningeal vessel walls of cerebrum and cerebellum. **Methods:** We sought to investigate the clinical manifestations, current different diagnostic tools, various therapeutic strategies and most uncommon subtypes of the disease. **Results:** The vast majority of CAA remains sporadic, with increasing prevalence with age and very frequent coexistence with Alzheimer’s disease. Clinically, CAA can present with spontaneous lobar intracerebral hemorrhage, transient focal neurologic episodes attributed to convexity subarachnoid hemorrhage or cortical superficial siderosis, and progressive cognitive decline leading to dementia. Inflammatory CAA subtype should be recognized early and treated promptly so that better functional outcomes may be achieved. Moreover, genetic and iatrogenic CAA forms are rare, yet increasingly recognized during the last years. Therapeutic management remains challenging for clinicians, especially when markers indicative of higher bleeding risk are present. A targeted therapy does not currently exist. However, various clinical trials are in progress, focusing on offering new promising insights into the disease treatment. **Conclusions:** This review aims to deepen our understanding of CAA diagnosis and therapeutic approach but also summarizes current evidence on the most uncommon subtypes of this cerebral small-vessel disease.

## 1. Introduction

Sporadic Cerebral Amyloid Angiopathy (CAA) is the most common form of CAA, characterized by a progressive vascular deposition of amyloid-β (Aβ) in small, cortical, and leptomeningeal vessels [1]. CAA is a disease of middle-aged or older individuals. However, asymptomatic neuropathological changes can begin approximately 30 years before the first hemorrhagic lesions manifest [1,2,3]. In this context, cognitive impairment may procced the hemorrhagic complications [3]. Sporadic CAA represents one of the most common forms of cerebral small vessel disease and is a major cause of non-traumatic lobar intracerebral hemorrhage (ICH), accounting for 12% of cases with spontaneous ICH cases [2,4]. Furthermore, it should be noted that Alzheimer’s disease (AD) pathology co-exists with CAA in approximately 85% of cases [5,6].

The clinical manifestations, combined with characteristic hemorrhagic and/or non-hemorrhagic findings on imaging, enable the in vivo diagnosis of possible or probable CAA, according to the pathologically validated Magnetic Resonance Imaging (MRI)-based Boston criteria, limiting the necessity for more invasive diagnostic methods, i.e., brain biopsy (Table 1) [7,8,9,10]. Nevertheless, for the definite diagnosis of the disease, histopathological confirmation is required.

**Figure 1:**
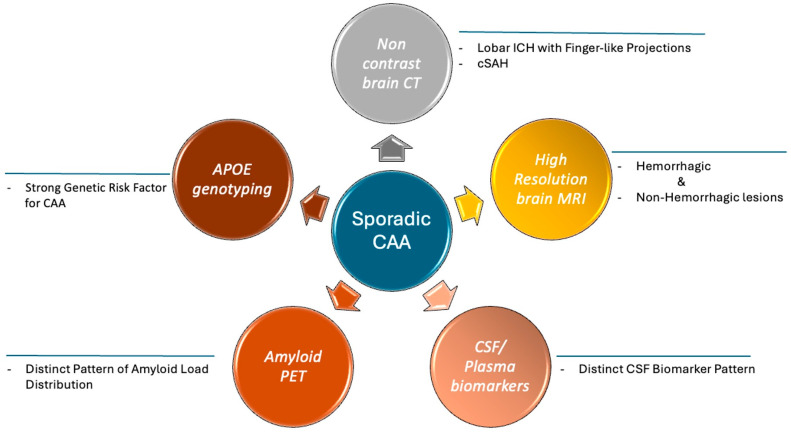
Useful tools in the diagnosis of Cerebral Amyloid Angiopathy.

Cerebrospinal fluid (CSF) and/or plasma biomarkers, in combination with brain MRI, amyloid positron emission tomography (PET), and Apolipoprotein E (APOE) genotyping may assist in the earlier recognition of the disease and the detailed workup of patients with cognitive impairment (Figure 1) [12,13,14]. On the other hand, the management of CAA focuses on the prevention of cognitive deterioration and the mitigation of the hemorrhagic risk.

Early-onset types of CAA, resulting from inflammatory, genetic, or iatrogenic causes, have been increasingly recognized in recent years [15]. These causes warrant specific attention with respect to a timely diagnosis, management, and genetic guidance, if required. Although rare, diagnosis of the inflammatory subtype of CAA remains an important clinical challenge, since early recognition and prompt treatment initiation may improve disease prognosis [16].

This narrative review aims to assess and summarize the most current literature on the pathophysiology, the clinical and neuroimaging manifestations, as well as the early-onset and rare types of the disease. We will also highlight recent diagnostic criteria, therapeutic strategies, and future perspectives for ongoing clinical research and practice.

## 2. Pathophysiology and Progression of the Sporadic CAA

In CAA, amyloid-β accumulation is detected primarily in the neocortical and leptomeningeal arterioles, and to a lesser extent in capillaries and venules [17,18]. The CAA-related vascular amyloid is composed of the 40-amino acid (Aβ40) fragment, suggesting a different underlying pathophysiological mechanism in comparison to AD pathology, where the amyloid plaques are predominantly composed of the 42-amino acid (Aβ42) residue fragment [19]. The factors that predispose or affect the Aβ deposition in sporadic CAA are not well understood. Transgenic mouse models have suggested various possible underlying mechanisms. Key-role in the evolution of CAA plays the impairment of the perivascular drainage system, leading to an aging brain due to a progressively increasing Aβ entrapment and deposition in the walls of small arteries [17,20]. Impairment of the perivascular drainage is also believed to be related to the frequently detected and characteristic dilation of perivascular spaces (known as Virchow–Robin spaces) on high-resolution brain MRI, found not only in lobar but also in deeper white matter regions (e.g., the centrum semiovale) [21,22,23]. APOE has also emerged as a crucial genetic factor, playing a significant role in Aβ metabolism, aggregation, and clearance [24,25].

Recently, four stages of the disease have been proposed, outlining the timeline from subclinical pathology to the most severe clinical manifestations [3,26]. The evolution of these stages spans approximately two to three decades. Initial vascular amyloid deposition represents the first step of this process, followed by alterations in the cerebrovascular unit during the second stage [3]. These alterations include loss of smooth muscle cells, vessel wall thickening with associated lumen restriction, and endothelial dysfunction [27,28]. In stage three, these alterations lead to fragile vessels and non-hemorrhagic brain injury, manifested as cerebral microinfarcts and white matter changes, with cognitive impairment being its prominent clinical manifestation [29]. In stage four, different hemorrhagic brain lesions appear, representing the most severe stage of the disease [30].

## 3. Diagnosis

### 3.1. Clinical Manifestations

Patients with CAA present with various clinical manifestations. A recent systematic review and meta-analysis showed that mild cognitive impairment (MCI)/dementia, transient focal neurological episodes (TFNEs), and focal neurological deficits from ICH are the most common clinical features, affecting 50% of patients with CAA [5]. Slower processing speed and worse executive function are the main affected cognitive domains in CAA, and this is associated with lower total brain volume, similarly to the non-CAA vascular cognitive impairment [31,32]. Neuroimaging markers, including cerebral cortical microinfarcts, lobar ICH, and disseminated cortical superficial siderosis (cSS), have been associated with worse cognitive performance [33,34]. Nevertheless, dementia in CAA is considered to be mediated by AD pathology as well, since those two neuropathologies coexist in the majority of cases [35,36,37].

CAA-related TFNEs, previously referred also as “amyloid spells”, are recurrent, stereotyped, and brief (usually lasting few minutes) episodes associated with disturbances in motor, sensory, visual, or language functions [38,39]. Recurrent TFNEs are mainly observed among CAA patients with either convexity subarachnoid hemorrhage (cSAH) or cSS, which represent the radiological result of a chronic cSAH [9,40]. cSAH and/or cSS presence or evolution might be a potential biomarker for assessing disease severity and future ICH risk, defining the management of these patients with regard to antiplatelets/anticoagulants as well [41].

Nevertheless, the most widely recognized clinical/radiological manifestation of both sporadic and hereditary CAA remains the intracerebral macrohemorrhage, typically with a diameter larger than 1 cm, located in the lobar and superficial cerebral and/or cerebellar cortex [30,42].

### 3.2. Neuroimaging Findings

**Figure 2:**
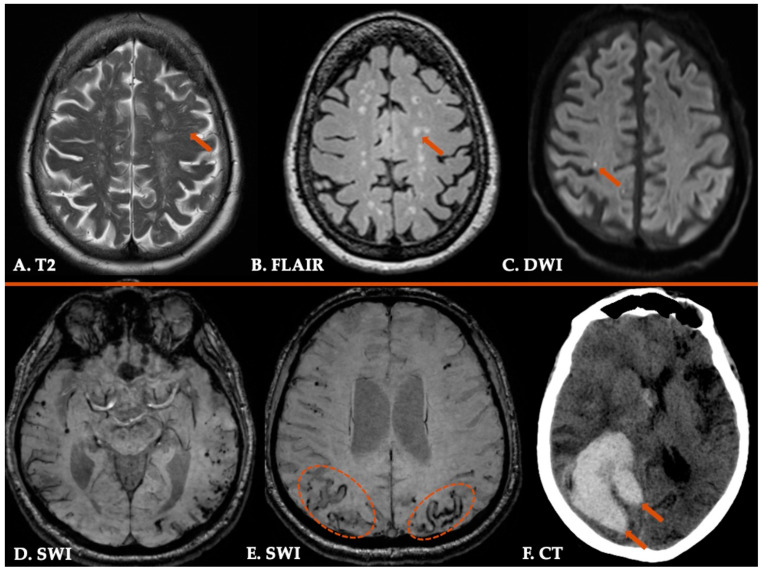
Neuroimaging findings in a patient with Cerebral Amyloid Angiopathy. Enlarged perivascular spaces in centrum semiovale (Panel (**A**)) and characteristic multispot white-matter hyperintensities pattern (Panel (**B**)) are detected in T2-weighted image and 3D fluid-attenuated inversion recovery sequence (FLAIR), respectively. Diffusion-weighted imaging (DWI) sequence (Panel (**C**)) showing a right parietal cortical microinfarct. Susceptibility-weighted imaging (SWI) sequences demonstrating multiple cerebral lobar microbleeds (Panel (**D**)) and disseminated cortical superficial siderosis (Panel (**E**)) in parietal lobes bilaterally. Fourteen months following CAA diagnosis, the patient presented focal neurological deficits attributed to a lobar hemorrhage of the right temporal lobe with characteristic finger-like projections (Panel (**F**)).

Common neuroimaging features among patients with CAA, indicative of small vessel disease, include hemorrhagic and non-hemorrhagic markers (Figure 2) [1]. The regional distribution of hemorrhagic lesions has been already described with a preferential clustering in the occipital and temporal lobes [43]. The Edinburgh or simplified Edinburgh computed tomography (CT) criteria, which focus on ICH morphology on CT imaging and combine the presence of subarachnoid hemorrhage (SAH) with finger-like projections (defined as extensions from the ICH that are longer than wide), are highly specific for identifying moderate/severe CAA, whereas the presence of at least one of these two markers was shown to have reasonable diagnostic sensitivity [44,45].

However, the gold-standard neuroimaging modality for the diagnosis of CAA remains the high-resolution brain MRI, with a protocol incorporating T2-weighted sequences, fluid attenuated inversion recovery (FLAIR) sequences, T2*-weighted axial sequences (conventional T2*-gradient recalled-echo or the more sensitive susceptibility-weighted imaging sequences, on 1.5 or preferably 3.0 Tesla MRI scanners) and diffusion-weighted imaging [38,46]. A recent meta-analysis showed that lobar ICH, cSS, and cerebral microbleeds (CMBs) are all common findings among patients with CAA [7]. The location of CMBs is more frequent in the occipital compared to the frontal or parietal cortex [43]. Non-aneurysmal cSAH, limited to the convexities of the brain, is attributed, in the majority of patients aged > 60 years, to underlying CAA [47,48,49]. cSAH represents the prodromal or acute phage/stage of cSS and may be clinically manifested with TFNEs [50]. Visible enlarged perivascular spaces in the centrum semiovale detected in T2-weighted sequences and white matter hyperintensities in a multispot pattern detected in FLAIR sequences represent also common neuroimaging findings and were incorporated in the recently revised Boston criteria for the diagnosis of CAA, enhancing their sensitivity [10,23,51,52].

Despite the fact that cerebral cortical microinfarcts (CMI) have not been included in the revised Boston criteria v2.0 for the diagnosis of CAA, they are considered a new neuroimaging marker of CAA pathology [53]. CMIs, with a diameter of 1–2 mm, are detected mainly in 3T MRI scans, and their radiological characteristics, including the lesion size (larger lesion ≥ 5 mm in diameter are associated with microembolism, whereas smaller lesions are related to CAA) and the lesion location (the majority of CAA-related CMIs are restricted to cortical layer) may differentiate CMIs related to CAA from those attributed to microembolism [33,54]. In addition, strictly superficial distribution of cerebellar microbleeds and cSS have been associated with clinicoradiologically and pathologically proven CAA but also with increased global amyloid deposition on Pittsburgh Compound B PET (PIB-PET) [55,56]. These observations may differentiate CAA-related cerebellar ICH from hypertensive arteriopathy-related cerebellar ICH, located mostly in deep areas of the cerebellum [57].

### 3.3. Cerebrospinal Fluid and Plasma Biomarkers

CSF molecular biomarkers have emerged as a promising tool for the investigation/diagnosis of pre-symptomatic CAA and may assist in differentiating CAA from AD in cognitively impaired individuals [58]. In accordance with previously published cohort studies, recent systematic reviews and meta-analyses found that CSF biomarkers follow a distinct pattern, characterized by decreased levels of Aβ40 (amyloid-β) compared to healthy controls (HC) and patients with AD, and decreased levels of Aβ42 compared to HC but not compared to AD [12,59,60,61,62,63]. Moreover, the CSF ratio Aβ42/Aβ40 appears decreased in CAA compared to HC; however, it is comparable between CAA and AD patients. Interestingly, total tau and p-tau CSF levels are significantly higher in CAA compared to HC but lower compared to AD [12].

On the other hand, only scarce evidence exists regarding plasma biomarkers among patients with CAA. A recent meta-analysis suggested that plasma Aβ40 and Aβ42 levels cannot sufficiently discriminate CAA from healthy controls (HC) [12]. Unfortunately, further comparisons could not be made due to lack of data. Plasma p-tau 217 has also been recently suggested as a non-invasive biomarker, with a superior diagnostic performance over other plasma Aβ biomarkers for differentiating CAA from AD [64]. According to the currently available literature, plasma biomarkers remain unreliable in distinguishing sporadic CAA from AD and HC; therefore, larger studies are needed to address these associations.

### 3.4. PET Findings

PET imaging with ^11^C-Pittsburgh compound B (^11^C-PiB) or ^18^F-florbetapir tracers has also been studied and suggested as another tool for an earlier and more accurate diagnosis of CAA [65]. Unfortunately, it is known that vascular amyloid deposition, a hallmark of CAA, cannot be discriminated from the parenchymal amyloid deposition, characteristic of AD, on amyloid-PET [13].

However, evidence from preliminary cohort studies demonstrate a distinct pattern of amyloid load distribution, with higher global amyloid load in CAA compared to patients with hypertension-related ICH and HC but significantly lower compared to AD [66,67,68,69,70,71,72,73,74,75,76,77,78,79,80,81]. In addition, higher occipital amyloid deposition is detected in amyloid-PET among patients with CAA compared to those with AD [82]. These findings align with postmortem studies that identify the occipital cortex as the most frequently and severely affected brain region in CAA, while the frontal, parietal, and temporal lobes exhibit comparatively lesser involvement [83].

Recent studies emphasize the potential diagnostic role of amyloid-PET in identifying presymptomatic CAA. While prior research has primarily focused on whole cortex amyloid uptake, regional analyses, such as occipital-to-global ratios, may exhibit a higher diagnostic accuracy [14]. A negative amyloid-PET scan is particularly useful for excluding advanced CAA, especially when compared to HC and individuals with hypertensive ICH [84]. This knowledge could also contribute to a more accurate and safe identification of patients eligible for anti-amyloid β monoclonal antibody treatments [3].

### 3.5. Apolipoprotein E Gene

The APOE gene, located on the long arm of human chromosome 19, has been identified as the most significant and prevalent genetic risk factor for CAA [85,86]. Three codominant alleles, ε2, ε3, and ε4 have been identified, encoding different isoforms of the APOE protein, whereas the prevalence of the different alleles varies [87]. The APOE ε3 is the most prevalent, whereas the APOE ε2 is considered to be the rarest allele [24].

The APOE gene encodes for the APOE protein, which is one of the major plasma apolipoproteins and exists in three different isoforms: APOE2, APOE3, and APOE4. APOE isoforms contribute to different brain cell functions, including the integrity and morphology of the synapses, the amyloid β deposition, and the functionality of neurovascular units such as the brain–blood barrier [88,89].

The existing literature suggests that APOE can influence the transition of Aβ from a nontoxic, monomeric state to a toxic bioproduct, forming oligomers and protofibrils, while the magnitude of this effect of the different APOE isoforms varies [25]. APOE4 is considered to be detrimental to Aβ metabolism, APOE3 neutral, and APOE2 protective [90]. Despite that APOE2 has been associated with the inhibition of Aβ oligomers and protofibrils deposition, APOE2 is related to increased vascular fragility and bleeding [91,92]. Consequently, the ε2 allele has been associated with a more severe CAA type and a higher risk of ICH [93,94]. Similarly, APOE4 has also been associated with increased risk of sporadic CAA and CAA-related cerebral micro- or macrohemorrhages, as well as with increased risk of a more severe CAA pathology [93,95]. Homozygous APOE4 carriers have the highest risk of CAA pathology, the highest disease severity, and the highest risk of developing amyloid-related imaging abnormalities (ARIA) following Aβ immunotherapy [95,96,97]. Moreover, the ε4 genotype, and especially ε4 homozygosity, has also been associated with the presence of cSS and the risk of worse cognitive impairment [98,99].

## 4. Management

### 4.1. Management of Cognitive Impairment

Cognitive decline represents a prodromal clinical manifestation of CAA and a characteristic symptom of the non/pre-hemorrhagic stage of the disease [3]. Beyond CAA, age-related comorbidities, including AD and hypertension related arteriosclerotic small vessel disease could also substantially contribute to the cognitive dysfunction [100].

The aggressive management of vascular risk factors, including arterial hypertension, hyperlipidemia etc., may delay the progression of cognitive impairment. Acetylcholinesterase inhibitors (donepezil, rivastigmine, galantamine) and the NMDA receptor antagonist memantine have been widely used for the symptomatic AD [101]. Existing evidence shows only modest cognitive benefits for CAA patients, without significant impact on functional outcomes [102,103]. Nevertheless, some benefit has been proposed by single case reports, especially when an AD pathology co-exists [104]. Considering their relatively safe side-effect profile, the lack of other therapeutic options, and the progressive deterioration of this disease, treatment with any of these agents could be discussed in early stages of cognitive impairment of patients suspected to have both pathologies.

### 4.2. Management of Intracerebral Hemorrhage

The recommendations for ICH management in the acute phase among patients with underlying CAA pathology remain identical with those for the management of patients with non-CAA spontaneous ICH [105,106]. In brief, the first step in acute ICH management includes the implementation of all required actions to prevent or restrict hematoma expansion. Swift cessation of previous antiplatelets/anticoagulants use, administration of anticoagulation reversal (if indicated), acute blood pressure lowering and close monitoring, management of perihematomal edema and prevention of possible complications, such as thromboembolism or aspiration pneumonia, are of paramount importance in the setting of acute ICH management [107,108,109,110,111,112,113,114,115,116,117,118,119,120,121]. Antiepileptic medications are indicated in patients with ICH who present either with clinical seizures, or impaired consciousness and confirmed electrographic seizures [122,123]. Craniotomy, minimally invasive surgery, or ventriculostomy may be considered as hematoma evacuation measures, taking into account international guidelines and recommendations, with the aim to reduce mortality and maybe improve functional outcomes [124,125].

The individual risk of ICH recurrence should also be assessed. Increased risk of recurrent ICH has been observed among CAA-related ICH survivors, especially if cSAH and disseminated cSS have been detected [126,127]. This information should be taken into account so that tighter control of blood pressure, and restrictions in antiplatelet/anticoagulant use would be implemented, whereas at the same time alternative therapeutic approaches of atrial fibrillation management (if present) should be discussed. Moreover, non-steroidal anti-inflammatory agents should be avoided since they may increase the risk of recurrent intracerebral bleeding, while caution is warranted regarding the excessive lowering of low-density lipoprotein levels (<70 mg/dL), since it may also be associated with ICH recurrence [128,129,130,131,132].

### 4.3. Management of Transient Focal Neurological Episodes

Despite the absence of controlled trials and although the episodes may be self-limited, antiepileptic medications, including levetiracetam, topiramate and lacosamide, have been proposed for the management of TFNEs, with a satisfactory response [38]. Taking into account that these TFNEs may represent the acute manifestation of a cSAH, the previously reported therapeutic strategies in the setting of acute ICH management (including tight blood pressure control and antiplatelet/anticoagulant medication withdrawal) should be implemented [38,105]. Interestingly, case reports have described termination of amyloid “spells” after antiplatelet therapy discontinuation [133].

### 4.4. Management of Acute Ischemic Stroke: Intravenous Thrombolysis

The risk–benefit ratio of intravenous thrombolysis (IVT) in the acute ischemic stroke (AIS) setting among patients with CAA remains unknown, and there is conflicting evidence regarding the functional outcome, the risk of hemorrhagic transformation and mortality. CMBs burden, regardless of the underlying pathology and etiology, has emerged as an independent risk factor, which may increase the risk of sICH among patients with AIS treated exclusively with IVT [134]. Pairwise and individual-patient data meta-analyses showed that high CMB burden (>10 CMBs), identified on pretreatment brain MRI, increased the risk of sICH, parenchymal hematoma, and remote parenchymal ICH, compared to lower prevalence of CMBs (≤10) [134,135]. Higher CMB burden also increased the rates of 3-month poor functional outcome and mortality [135]. Based on this evidence, the European Stroke Organization (ESO) guidelines of IVT for AIS, recommended against IVT administration in patients with high CMB (>10) burden [136]. This recommendation is in accordance with the results of a prespecified post hoc analysis of the randomized controlled WAKE-UP clinical trial, showing increased odds of excellent functional outcome, despite a trend towards higher rates of bleeding complications, among patients with only moderate numbers of CMBs [137]. Although depiction of CMBs is impossible on non-contrast CT brain, relevant ESO guidelines recommended against systematic screening with brain MRI due to IVT delays [136].

Limited data exist regarding the safety and effectiveness of IVT among patients with previous lobar ICH and/or known cSS. Therefore, an evidence-driven recommendation could not be made during the development of the ESO guidelines [136]. Moreover, since CAA cannot be considered as a non-recurrent or treatable cause of brain hemorrhage, IVT should be administrated with caution even if an adequate, long time period has elapsed since the initial lobar ICH [136]. Similarly, no relevant recommendation can be formulated with regard to cSS preexistence and the risk of bleeding after IVT, since the prevalence of cSS among AIS patients who received IVT has been reported only in limited cohorts [138,139]. Nevertheless, the evidence shows increased risk of remote parenchymal hemorrhage coupled with clinical deterioration [139].

### 4.5. Management of Acute Ischemic Stroke: Mechanical Thrombectomy

Limited data exist on the safety and effectiveness of endovascular thrombectomy with or without IVT among patients with known or underlying CAA. Cohort studies revealed no increased risk of hemorrhagic complications and mortality following mechanical thrombectomy in the AIS setting, despite the higher rates of worse functional outcome at 3 months [140,141,142].

### 4.6. Secondary Prevention and Left Atrial Appendage Occlusion

The use of antithrombotic agents for secondary prevention among patients with hemorrhagic lesions suggestive of CAA remains a significant challenge for the clinicians. Based on the evidence from large clinical trials, the guideline-based antiplatelet therapy for secondary prevention among patients with ischemic stroke or myocardial infarction should not be influenced by the number of CMBs at baseline [143,144,145]. Moreover, data on cSS and antithrombotic therapy are limited and of uncertain quality, whereas dual antiplatelet therapy, as an individualized approach, should be probably avoided in patients with disseminated cSS [41,146].

CMIs represent another significant challenge for the clinicians. These DWI hyperintensity lesions seem to share similar underlying pathophysiological mechanisms with hemorrhagic lesions, like CMBs. Both are attributed to the ruptured leptomeningeal and cortical fragile vessels due to amyloid deposition [147,148]. Moreover, CMIs have been strongly related to cSS, since a close spatial association between CMIs and cSS has been reported [149]. Consequently, clinicians should be cautious when administering antiplatelets or anticoagulants in CAA patients, presenting with CMIs, since their underlying mechanism is distinct from that of microembolic lesions.

Among CAA patients without previous ICH, who have an indication for anticoagulation due to underlying atrial fibrillation or venous thromboembolism, anticoagulant treatment, preferably with apixaban may be suggested [150,151]. However, the presence of disseminated cSS should be considered as a contraindication for anticoagulation [127,152]. A reduced dose of DOACs (apixaban 2.5 mg BID, or dabigatran 110 mg BID or rivaroxaban 15 mg) has also been proposed as an individualized approach in patients with excessive bleeding risk. However, the existing evidence is not adequate to support this suggestion, especially in patients with normal renal clearance [153,154].

Prior ICH history makes the decision for anticoagulation even more challenging [155]. COCROACH collaboration, an individual patient data meta-analysis of randomized-controlled clinical trials, comprising 103 participants with lobar ICH or non-aneurysmal cSAH found no interaction between ICH location and the effects of oral anticoagulation [156]. On the other hand, the Data Monitoring Committee of the ENRICH-AF trial decided to terminate the enrolment of patients with lobar ICH and cSAH, after reviewing the safety data of the first 699 patients [157]. The Committee observed unacceptably high risks of recurrent hemorrhagic stroke among patients with lobar ICH and cSAH, assigned to the edoxaban arm, leading to this decision. Currently, the data from four ongoing or completed clinical trials are expected to guide the management of this specific patient subgroup [158].

Percutaneous left atrial appendage occlusion (LAAO) may represent a very promising alternative in the management of CAA patients with underlying atrial fibrillation. Limited data from small-scale cohort studies show low rates of periprocedural complications and a promising safety and effectiveness profile [159,160]. However, these observations should be confirmed in larger multicenter cohort studies.

## 5. CAA Mimics

**Figure 3:**
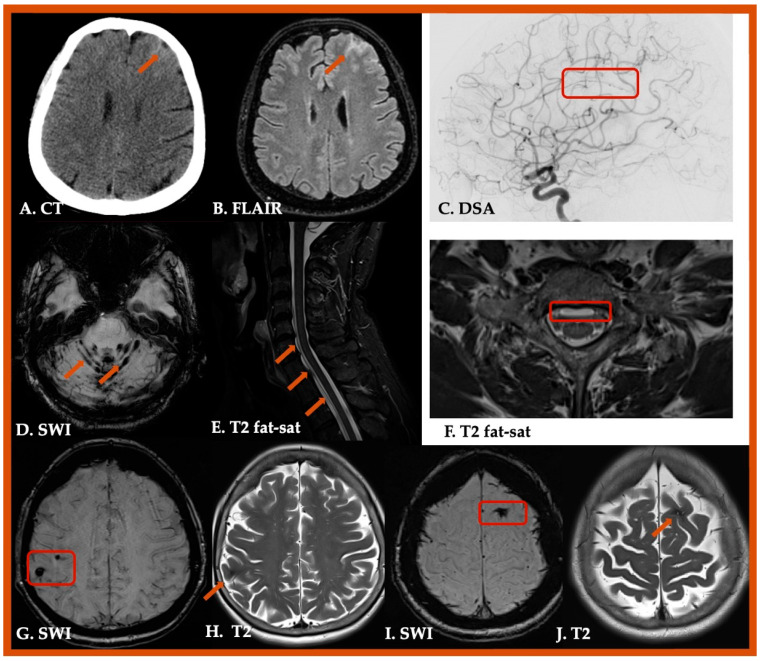
Radiological mimics of Cerebral Amyloid Angiopathy. A 68-year-old female patient with convexity subarachnoid hemorrhage, detected on brain Computed tomography (Panel (**A**)) and fluid-attenuated inversion recovery sequence (FLAIR) (Panel (**B**)), attributed to reversible cerebral vasoconstriction syndrome (RCVS) with characteristic vasoconstriction in the left pericallosal artery (Panel (**C**)). Another 58-year-old male patient with extensive infratentorial superficial siderosis (Panel (**D**), which was attributed to dural defect with extra-dural CSF, detected on T2-weighted fat saturated sequences. (Panels (**E**,**F**)). A 77-year-old female patient with multiple cortical and subcortical cerebral hemorrhagic lesions attributed to multiple cerebral cavernous malformations (Panels (**G**–**J**)).

**Figure 4:**
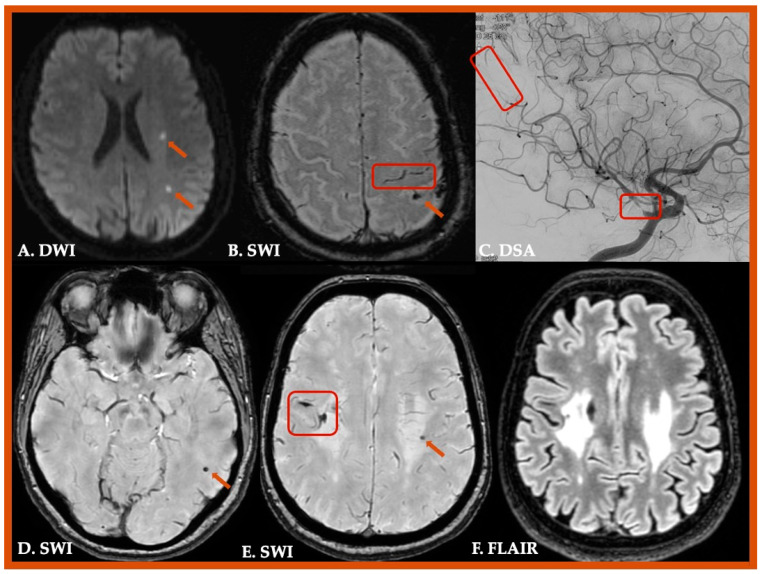
Radiological mimics of Cerebral Amyloid Angiopathy. A 55-year-old male patient with multiple cerebral microinfarcts (Panel (**A**)), left parietal cortical superficial siderosis and lobar microbleeds (Panel (**B**)) attributed to primary angiitis of the Central Nervous System. Digital subtraction angiography revealed multiple intracranial stenoses in both proximal and distal arteries (Panel (**C**)). A 57-year-old female patient with multiple lobar and deep hemorrhagic lesions (Panels (**D**,**E**)) associated with confluent, bilateral, symmetric white matter hyperintensities on FLAIR sequences, with relative sparing of subcortical U-fibers (Panel (**F**)). Genome wide sequencing revealed the pathogenic mutation NM_001845.6:c.2245G>A, p.(Gly749Ser), leading to the diagnosis of COL4A1-related disorder.

Particular attention is required to differentiate disorders which may resemble CAA [161]. CMBs could also be detected in other clinical conditions, such as primary or secondary central nervous system (CNS) vasculitis, CNS infections, infective endocarditis, cerebral fat embolism, radiation induced vascular malformations, hypoxemia, posterior reversible encephalopathy syndrome (PRES), COL4A1 small vessel arteriopathy, acute hemorrhagic leukoencephalitis (Weston–Hurst syndrome), or intravascular lymphoma [162,163,164,165,166,167,168,169,170,171,172,173,174,175]. Similarly, cSAH and/or cSS could be identified in various disorders, including reversible cerebral vasoconstriction syndrome (RCVS), cerebral venous thrombosis, and moyamoya syndrome [176,177,178]. Characteristic cases of CAA mimics are illustrated in Figure 3 and Figure 4, and conditions mimicking CAA are summarized in Table 2.

## 6. Rare or Early-Onset Types of CAA

### 6.1. Cerebral Amyloid Angiopathy-Related Inflammation (CAA-ri)

Cerebral Amyloid Angiopathy-related inflammation (CAA-ri) represents a rare, albeit distinct, subtype of CAA [179,180,181]. CAA-ri is characterized by the predominant amyloid-Aβ deposition in the media and adventitia of cortical and leptomingeal cerebral vessels, with an associated characteristic perivascular nondestructive accumulation of inflammatory cells [180,182]. The precise pathophysiology and progression of the disease remains unknown. The most prevalent hypothesis involves an inflammatory autoimmune response against cerebrovascular Aβ-amyloid deposits [180,182]. CAA-ri represents a disease common among the elderly, with an average age of 67–71 years at diagnosis, without apparent gender predominance [16,180,183]. Based on the diagnostic criteria proposed by Auriel et al., which achieved a sensitivity of 82% and specificity of 97% for the probable diagnosis, CAA-ri is becoming nowadays more widely identified (Table 1) [11]. The greater availability of high-resolution MRI plays also a critical role, allowing a prompt and reliable diagnosis, and bypassing invasive diagnostic methods, such as brain biopsy [11,16].

**Figure 5:**
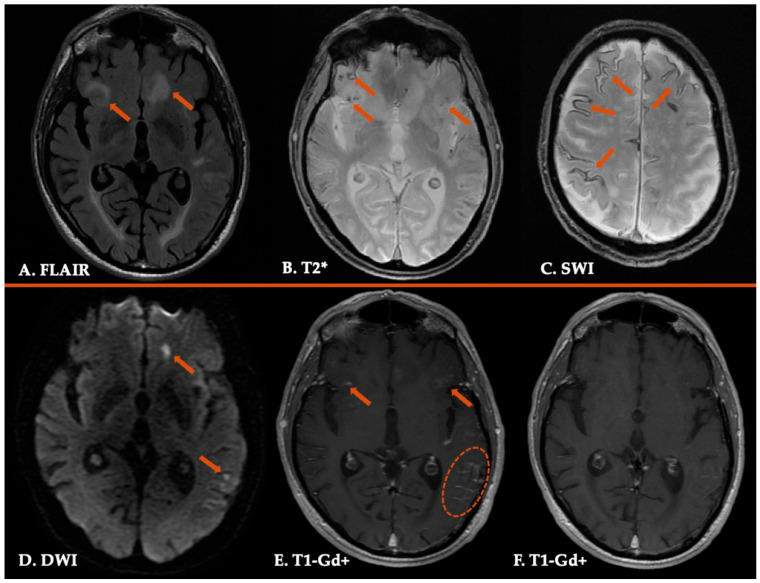
Neuroimaging findings in a patient with Cerebral Amyloid Angiopathy-related inflammation. Three-dimensional fluid-attenuated inversion recovery sequence (FLAIR) revealing multiple bilateral asymmetric white matter hyperintensities (Panel (**A**)). Axial T2* images showing multiple lobar cerebral microbleeds (Panel (**B**)) and disseminated cortical superficial siderosis (Panel (**C**)). Diffusion-weighted imaging (DWI) sequence (Panel (**D**)) demonstrating multiple cortical and subcortical ischemic lesions (Panel (**D**)). T1-weighted imaging showing extensive leptomeningeal gadolinium enhancement (Panel (**E**)) with complete resolution following intravenous treatment with methylprednisolone (Panel (**F**)).

CAA-ri presents with a variety of clinical manifestations, including cognitive decline as the most prevalent, focal neurological deficits, encephalopathy, headache, epileptic seizures and psychiatric symptoms [16]. The most common neuroimaging markers (Figure 5) include the characteristic bilateral, asymmetric, confluent T2/FLAIR hyperintense white matter lesions, associated with multiple lobar microbleeds and leptomeningeal gadolinium enhancement [16,184,185,186,187]. cSS, lobar macro-hemorrhage, cSAH and cortical ischemic lesions represent less frequent radiological findings of this entity [16,187,188]. Other, less frequent neuroimaging features associated with CAA-ri are the sulcal hyperintensities and gyral swelling [189].

Interestingly, CMBs in CAA-ri are detected mainly colocalizing with the T2/FLAIR hyperintense white matter lesions and their distribution does not follow the occipital predominance pattern, described in CAA [190,191]. Moreover, the incidence of multiple CMBs in CAA-ri is much higher compared to CAA, whereas susceptibility weighted imaging has higher sensitivity than T2*-weighted gradient echo imaging for the detection of CMBs [10,192,193]. On the other hand, lobar macro-hemorrhages and focal or disseminated cSS are not commonly detected in the initial presentation of CAA-ri compared to CAA [185].

Amyloid beta related angiitis (ABRA) is characterized by a destructive, vasculitic, transmural, mainly granulomatous, inflammatory infiltrate, and probably represents part of a more severe spectrum of the same underlying entity [194,195,196]. ABRA and CAA-ri cannot be distinguished based on clinical and neuroimaging findings [194,195,196]. Their distinction is possible only at the neuropathological level [163].

Unfortunately, there are no data from randomized-controlled clinical trials investigating therapeutic options for CAA-ri. Evidence from observational studies suggest that immunosuppression may be beneficial, especially when initiated early [197]. Corticosteroids represent the first-line treatment in patients with CAA-ri and have been associated with clinical and radiological recovery from the primary episode and a reduced relapse risk [193,197,198,199]. Additional immunosuppressive therapies, including cyclophosphamide, mycophenolate mofetil, azathioprine, immunoglobulin or rituximab have been used as secondary-line therapies for cases of severe progression or repeated relapses [197,200]. Brain biopsy should be considered for patients who fail to respond to high-dose corticosteroid treatment or experience recurrent relapses [193,197,201].

Following the initial high-dose corticosteroid treatment, the clinical and radiological manifestations may be substantially mitigated, whereas in many cases the disease has a single-phase course with favorable recovery [202]. However, relapses can be observed after withdrawal of steroids or during tapering. The probability of CAA-ri recurrence differs across various cohort studies; interestingly, increased CMBs may be detected in T2*/SWI sequences following disease relapses [193,197,202]. Clinicians should be aware of this condition since a timely diagnosis and swift initiation of immunosuppressive therapy may improve the disease prognosis.

### 6.2. Hereditary CAA

Rare monogenic disorders represent another type of early-onset CAA [15]. This category includes mutations (such as the Dutch mutation) involving the amyloid-β coding domains of the amyloid precursor protein gene (APP), mutations primarily associated with familial AD (where CAA can also co-exist; copy number variants of APP, including duplications or triplications like trisomy 21, mutations of PSEN1 and PSEN2), and non-amyloid-β CAA mutations like mutations in the integral membrane protein 2B (ITM2B), cystatin C or gelsolin amyloidosis, and mutations in transthyretin [11,203,204,205,206,207,208].

Clinical manifestations include migraine, recurrent strokes (mainly ICH, and ischemic), seizures, cognitive decline and dementia in patients with APP mutations [203,209]. Patients with PSEN1 and PSEN2 mutations present with familial AD with a phenotype that is characteristically amnestic, but can also include seizures, psychosis, myoclonus, spastic paraparesis, extrapyramidal and cerebellar signs [204,210,211]. Very early ICH, typically before the age of 40 associated with a severe progressive neurological deterioration due to multiple strokes and an average life expectancy of approximately 30 years, represents the clinical features of patients with cystatin C mutations [212,213]. Additionally, leptomeningeal involvement and recurrent subarachnoid hemorrhages could be the prominent clinical presentation of patients with mutations in transthyretin [214].

Genetic testing, either via neurodegenerative panel or whole exome, or even whole genome sequencing (WES and WGS, respectively), is recommended when there is clinical or radiological suspicion of early-onset CAA, with or without supporting familial history and the prognosis differs among the various genetic variants. Unfortunately, there is no available targeted treatment for hereditary CAA nowadays, and all conservative management approaches, already discussed in the sporadic CAA, could also be applicable in these patients.

### 6.3. Iatrogenic CAA

Iatrogenic CAA has been recently described among younger patients who developed CAA, 2 or 3 decades following neurosurgical procedures earlier in childhood [215]. The iatrogenic form of CAA was suspected to be caused by a prion-like transmission of Aβ from cadaveric material, including cadaveric dural grafts (cadaveric dura), or embolization material, or surgical instruments, or cadaveric human growth hormone [216,217,218,219]. Diagnostic criteria have been proposed, and recently modified, aiming to standardize the research on iatrogenic CAA [220,221]. Interestingly, based on limited case reports, past history of red blood cell transfusion among patients with CAA has also been recently considered as possible means of iatrogenic CAA transmission [222,223,224]. Future systematic research is required to investigate whether CAA could be transmissible through transfusion.

History of previous potential exposure, characteristic clinical and neuroimaging CAA features, evidence of amyloid-β accumulation in the CNS, documented either on amyloid PET-CT or on CSF biomarkers profile or on brain biopsy and exclusion of genetic causes of Aβ CNS disease should be investigated in cases with suspicion of iatrogenic CAA [221]. The presence of deep cerebral microbleeds can also be detected in more than 40% of patients with iatrogenic CAA during the follow-up period [221]. Moreover, mainly ipsilateral spread of hemorrhagic lesions and frequently detected inflammatory lesions have also been reported [225,226]. Evidence from small to moderate-sized cohorts indicates rapid and aggressive progression of hemorrhagic lesions with associated high risk of recurrent ICH but also with lower mortality risk [221]. Targeted therapies are presently unavailable for iatrogenic CAA, similar to other CAA variants.

## 7. Conclusions and Future Directions

In this narrative review we sought to provide an overview of the clinical and neuroimaging characteristics of CAA, the key components of the diagnostic evaluation and the disease management. Moreover, we shortly discussed significant aspects of CAA-ri, a rare but distinct and potentially treatable subtype of CAA. We also highlighted the most uncommon subtypes of CAA including hereditary and iatrogenic CAA.

During the past decades significant progress in our comprehension of CAA has been achieved, involving the underlying pathophysiological mechanisms, the proposed means of Aβ deposition, the genetic causes of early-onset CAA, the outcomes associated with the use of different therapies, and the novel anti-amyloid therapies.

Increased vigilance is suggested with the use of anti-amyloid-β monoclonal antibodies, approved for AD-related MCI. APOE ε4 alleles and baseline presence of hemorrhagic lesions (CMBs and/or cSS), indicative of a coexisting CAA pathology, represent significant risk factors of developing amyloid-related imaging abnormalities [227]. The therapeutic potential of novel agents, including mivelsiran (NCT06393712), and the development of APOE-targeted therapies are currently under investigation [25]. Moreover, minocycline, an already widely used antibiotic agent, has been proposed for the treatment of severe CAA due to its possible effect on inflammation, gelatinase activity, and angiogenesis [228,229]. Notably, these trials differ substantially on study populations, eligibility criteria and safety and efficacy outcomes and their upcoming results should be evaluated with caution, taking into account the limited understanding of the underlying pathophysiological mechanism of sporadic and other CAA subtypes. Consequently, future research should aim to fully elucidate disease’s pathophysiology and larger-scale clinical trials are required to thoroughly assess the safety and efficacy of different agents. In conclusion, CAA represents an emerging disease entity with multiple manifestations and subtypes that is currently intensively investigated in order to develop individualized treatment approaches targeted to the underlying causative mechanisms.

## Figures and Tables

**Table 1:** Diagnostic criteria for Cerebral Amyloid Angiopathy and Cerebral Amyloid Angiopathy-related inflammation [10,11].

**Boston Criteria for the Diagnosis of CAA (Version 2.0)**	
**1. Definite CAA**	Full post-mortem examination demonstrating the following: • Presentation with spontaneous ICH, TFNEs, cSAH, or CI/Dementia • Severe CAA with vasculopathy • Absence of other diagnostic lesion
**2. Probable CAA with supporting** **pathology**	Clinical data and pathologic tissue (evacuated hematoma or cortical biopsy) demonstrating the following: • Presentation with spontaneous ICH, TFNEs, cSAH, or CI/Dementia • Some degree of CAA in specimen • Absence of other diagnostic lesion
**3. Probable CAA**	Clinical data and MRI demonstrating the following: • Age ≥ 50 years • Presentation with spontaneous ICH, TFNEs, or CI/Dementia • ≥2 of the following strictly lobar hemorrhagic lesions on T2*-weighted MRI, in any combination: ICH, CMB, cSS/cSAH foci OR • 1 lobar hemorrhagic lesion + 1 white matter feature (Severe CSO-PVS or WMH-MS) • Absence of any deep hemorrhagic lesions (ICH, CMB) on T2*weighted-MRI • Absence of other cause of hemorrhagic lesions * • Hemorrhagic lesion in cerebellum not counted as either lobar or deep hemorrhagic lesion
**4. Possible CAA**	Clinical data and MRI demonstrating the following: • Age ≥ 50 years • Presentation with spontaneous ICH, TFNEs, or CI/Dementia • Absence of other cause of hemorrhage * • 1 strictly lobar hemorrhagic lesion on T2*-weighted MRI: ICH, CMB, cSS/cSAH focus OR • 1 white matter feature (Severe CSO-PVS or WMH-MS) • Absence of any deep hemorrhagic lesions (ICH, CMB) on T2*-weighted MRI • Absence of other cause of hemorrhagic lesions * • Hemorrhagic lesion in cerebellum not counted as either lobar or deep hemorrhagic lesion
B. **Criteria for the Diagnosis of CAA-ri**	
**1. Definite CAA-ri**	Definitive diagnosis of CAA-ri requires brain biopsy.
**2. Probable CAA-ri**	1. Age > 40 y 2. Presence of ≥1 of the following clinical features: headache, decrease in consciousness, behavioral change, or focal neurological signs and seizures; the presentation is not directly attributable to an acute ICH 3. MRI shows unifocal or multifocal WMH lesions (corticosubcortical or deep) that are asymmetric and extend to the immediately subcortical white matter; the asymmetry is not due to past ICH 4. Presence of ≥1 of the following corticosubcortical hemorrhagic lesions: cerebral macrobleed, cerebral microbleed, or cortical superficial siderosis 5. Absence of neoplastic, infectious, or other cause
**3. Possible CAA-ri**	1. Age ≥ 40 y 2. Presence of ≥1 of the following clinical features: headache, decrease in consciousness, behavioral change, or focal neurological signs and seizures; the presentation is not directly attributable to an acute ICH 3. MRI shows WMH lesions that extend to the immediately subcortical white matter 4. Presence of ≥1 of the following corticosubcortical hemorrhagic lesions: cerebral macrobleed, cerebral microbleed, or cortical superficial siderosis 5. Absence of neoplastic, infectious, or other cause

* Other causes of hemorrhagic lesion: antecedent head trauma, hemorrhagic transformation of an ischemic stroke, arteriovenous malformation, hemorrhagic tumor, central nervous system vasculitis. Other causes of cSS and acute cSAH should also be excluded. Abbreviations: CAA: Cerebral Amyloid Angiopathy, ICH: intracerebral hemorrhage, TFNE: transient focal neurologic episodes, cSAH: convexity subarachnoid hemorrhage, CI: cognitive impairment, MRI: magnetic resonance imaging, cSS: cortical superficial siderosis, CSO-PVS: visible perivascular spaces in the centrum semiovale, WMH-MS white matter hyperintensities in a multispot pattern, CMB: cerebral microbleed, CAA-ri: Cerebral Amyloid Angiopathy-related inflammation.

**Table 2:** Summary of main conditions mimicking CAA, CAA-related inflammation, iatrogenic CAA, and hereditary CAA.

Conditions Mimicking CAA, CAA-ri, Iatrogenic CAA, and Hereditary CAA	
**CAA mimics**	Hypertension-related small vessel disease Multiple or familiar cerebral cavernous malformations Reversible cerebral vasoconstriction syndrome (RCVS) Cerebral venous thrombosis Vascular malformations (e.g., dural arteriovenous fistulas) Moyamoya syndrome Radiation induced vascular malformations Haemorrhagic metastases Critical illness, for example, due to severe COVID-19 infection Acute haemorrhagic leukoencephalitis (Weston–Hurst syndrome) Infective endocarditis Head trauma—diffuse axonal injury Intravascular lymphoma Cerebral venous thrombosis Cardiac myxoma
2. **CAA-ri mimics**	Primary Angiitis of Central Nervous System (PACNS) Posterior reversible encephalopathy syndrome (PRES) Delayed leucoencephalopathy as a complication after endovascular therapy of intracranial aneurysms Hereditary diffuse leukoencephalopathy with spheroids (HDLS) [mutations in the CSF1R (colony stimulating factor-1 receptor) gene] Cerebral Amyloidoma
3. **Iatrogenic CAA mimics**	Radiation induced vascular malformations Vasculitis of Central Nervous System Amyloid-related imaging abnormalities (ARIA)
4. **Hereditary CAA mimics**	COL4A1 small vessel arteriopathy Cerebral autosomal dominant arteriopathy with subcortical infarcts and leukoencephalopathy (CADASIL) Cerebral autosomal recessive arteriopathy with subcortical infarcts and leukoencephalopathy (CARASIL) Cathepsin-A Related Arteriopathy with Strokes and Leukoencephalopathy (CARASAL) Fabry disease Hereditary diffuse leukoencephalopathy with spheroids (HDLS) [mutations in the CSF1R (colony stimulating factor-1 receptor) gene]

## Data Availability

All data needed to evaluate the conclusions in the paper are present in the main manuscript. Additional data related to this paper may be requested from the corresponding author upon reasonable request.
